# Removing the economic burden of road traffic injuries from patients: a successful model

**DOI:** 10.5249/jivr.v7i2.744

**Published:** 2015-07

**Authors:** Hosein Karim, Mehdi Mohammadi, Shahrzad Bazargan-Hejazi, Alireza Ahmadi

**Affiliations:** ^a^Department of Cardiology, Imam Ali Hospital, Kermanshah University of Medical Science, Kermanshah, Iran.; ^*b*^Kermanshah University of Medical Science, Kermanshah, Iran.; ^*c*^Department of Psychiatry, College of Medicine, Charles Drew University of Medicine and Science & David Geffen School of Medicine at UCLA.; ^*d*^Department of Anesthesiology, Critical Care and Pain Management, Imam Reza Hospital, Kermanshah University of Medical Science, Kermanshah, Iran.

All costs for treatment of road crash patients are free in the Islamic Republic of Iran. In this model, Islamic Republic of Iran offers a synergistic approach that is efficient from both the cost and health outcome sides. In this system, all costs of treatment for victims of road crashes are free regardless of who was at fault in causing the crash. It is more than 10 years that insurance companies are mandated to deposit 10 percent of their total revenue from car insurances (Third Party Insurance) directly to the Department of Budget and Treasury in the Ministry of Health. Ministry of Health in return is obligated to cover the cost of medical care including hospital, emergency care facilities, and other care center, to car crash victims, without checking whether they carry any insurance or not. Therefore, patients are not liable for payment of cost. To have the cost of treatment waved injured, patients must submit a police report. However, delays in submitting such report are acceptable.

This system mandates public and private hospitals and other health care centers to provide uncompensated care to road crash victims without demanding any payment from them, their family, relatives, or the person who is legally at fault for a crash or personal injury. Private hospitals can be reimbursed by filling appropriate documentation to the Ministry of Health for all costs incurred by these patients. Hospitals or medical centers that deny treatment to road crash patients or obligate them to pay admission or treatment costs may face criminal penalties for withholding aid that could lead to further injury, illness, or death.

The no-fault care system in the Islamic Republic of Iran addresses social inequality by removing financial burden^1^ and narrows disparities and gaps in access to care among car crashes victims. It also promotes the idea that government has a major responsibility to offer an appropriate response to the road crash victims beyond educational interventions that often focus on human errors. ^2,3^ Other countries may look to this system of care as a successful model for implementation. 
